# Supplementary material for: Hot spot prediction in protein-protein interactions by an ensemble system
Source: BMC Syst Biol. 2018 Dec 31;12(Suppl 9):132. doi: 10.1186/s12918-018-0665-8 (PMC6311905; doi:10.1186/s12918-018-0665-8)
Supplement: Supplementary file 2 — All relevant MATLAB code. A simple Matlab implement of our predictor. (ZIP 19,742 kb) [file 12918_2018_665_MOESM2_ESM.zip › Additional file 2/Read ASA/ASA Property/ASEdb/1DANU.txt]

 NetSurfP - Protein Surface Accessibility and Secondary Structure Predictions

  

|  |  |  |
| --- | --- | --- |
|  |  | NetSurfP - Protein Surface Accessibility and Secondary Structure Predictions  Technical University of Denmark |

  


---

```
# For publication of results, please cite:
# A generic method for assignment of reliability scores applied to solvent accessibility predictions.
# Bent Petersen, Thomas Nordahl Petersen, Pernille Andersen, Morten Nielsen and Claus Lundegaard
# BMC Structural Biology 2009, 9:51 doi:10.1186/1472-6807-9-51
#
# Column 1: Class assignment - B for buried or E for Exposed - Threshold: 25% exposure, but not based on RSA
# Column 2: Amino acid
# Column 3: Sequence name
# Column 4: Amino acid number
# Column 5: Relative Surface Accessibility - RSA
# Column 6: Absolute Surface Accessibility
# Column 7: Z-fit score for RSA prediction
# Column 8: Probability for Alpha-Helix
# Column 9: Probability for Beta-strand
# Column 10: Probability for Coil
E G  1DANU                  1    0.925  72.829  -1.219   0.003   0.003   0.994
E E  1DANU                  2    0.652 113.887  -1.744   0.018   0.019   0.964
E P  1DANU                  3    0.443  62.848  -1.299   0.018   0.047   0.935
E L  1DANU                  4    0.385  70.530  -0.374   0.018   0.088   0.893
B Y  1DANU                  5    0.206  44.086  -0.056   0.019   0.141   0.840
E E  1DANU                  6    0.415  72.413  -0.937   0.021   0.279   0.699
E N  1DANU                  7    0.554  81.120  -0.976   0.021   0.279   0.699
B S  1DANU                  8    0.279  32.746  -0.582   0.019   0.141   0.840
E P  1DANU                  9    0.577  81.834  -0.501   0.018   0.088   0.893
E E  1DANU                 10    0.512  89.499   0.296   0.018   0.088   0.893
B F  1DANU                 11    0.066  13.186  -0.860   0.019   0.141   0.840
E T  1DANU                 12    0.310  43.011   0.564   0.019   0.141   0.840
B P  1DANU                 13    0.181  25.712  -0.165   0.064   0.216   0.721
E Y  1DANU                 14    0.312  66.717   0.068   0.064   0.216   0.721
E L  1DANU                 15    0.449  82.212   0.714   0.064   0.216   0.721
E E  1DANU                 16    0.552  96.452  -0.322   0.064   0.216   0.721
B T  1DANU                 17    0.100  13.912  -0.272   0.064   0.216   0.721
E N  1DANU                 18    0.371  54.373   0.211   0.066   0.296   0.638
B L  1DANU                 19    0.095  17.394  -0.577   0.021   0.279   0.699
B G  1DANU                 20    0.239  18.833  -1.176   0.018   0.088   0.893
E Q  1DANU                 21    0.370  66.064   0.215   0.018   0.047   0.935
B P  1DANU                 22    0.213  30.196  -0.892   0.004   0.138   0.858
E T  1DANU                 23    0.438  60.764   0.036   0.004   0.616   0.381
B I  1DANU                 24    0.106  19.628   0.339   0.001   0.900   0.099
E Q  1DANU                 25    0.389  69.493   0.999   0.001   0.959   0.040
B S  1DANU                 26    0.122  14.334  -0.026   0.001   0.959   0.040
E F  1DANU                 27    0.377  75.664   0.551   0.001   0.959   0.040
B E  1DANU                 28    0.262  45.824   0.052   0.001   0.900   0.099
E Q  1DANU                 29    0.459  81.942   0.007   0.004   0.420   0.576
E V  1DANU                 30    0.627  96.339  -1.289   0.004   0.138   0.858
E G  1DANU                 31    0.683  53.768  -0.530   0.005   0.045   0.951
E T  1DANU                 32    0.325  45.050  -0.220   0.005   0.336   0.660
E K  1DANU                 33    0.309  63.479   1.308   0.001   0.900   0.099
B V  1DANU                 34    0.030   4.688   0.606   0.001   0.959   0.040
B N  1DANU                 35    0.249  36.395   1.600   0.001   0.959   0.040
B V  1DANU                 36    0.020   3.135   1.317   0.001   0.959   0.040
B T  1DANU                 37    0.159  22.053   1.309   0.001   0.959   0.040
B V  1DANU                 38    0.024   3.658   0.711   0.001   0.900   0.099
E E  1DANU                 39    0.342  59.765   0.941   0.003   0.718   0.279
B D  1DANU                 40    0.134  19.295   0.092   0.004   0.197   0.799
B E  1DANU                 41    0.145  25.262  -0.032   0.018   0.047   0.935
E R  1DANU                 42    0.468 107.172  -0.806   0.018   0.088   0.893
E T  1DANU                 43    0.354  49.030  -0.867   0.052   0.084   0.864
E L  1DANU                 44    0.260  47.606  -1.972   0.052   0.084   0.864
E V  1DANU                 45    0.413  63.463  -1.248   0.052   0.084   0.864
E R  1DANU                 46    0.498 114.065  -0.952   0.019   0.141   0.840
E R  1DANU                 47    0.608 139.301  -1.406   0.018   0.088   0.893
E N  1DANU                 48    0.674  98.674  -0.902   0.018   0.019   0.964
E N  1DANU                 49    0.442  64.650  -2.222   0.018   0.019   0.964
E T  1DANU                 50    0.480  66.562  -0.353   0.021   0.279   0.699
E F  1DANU                 51    0.370  74.319  -0.468   0.004   0.616   0.381
B L  1DANU                 52    0.175  32.079   0.716   0.021   0.756   0.223
B S  1DANU                 53    0.207  24.284  -0.370   0.021   0.756   0.223
B L  1DANU                 54    0.092  16.845  -0.310   0.021   0.756   0.223
E R  1DANU                 55    0.320  73.166  -0.140   0.021   0.756   0.223
B D  1DANU                 56    0.185  26.630  -1.443   0.018   0.846   0.136
B V  1DANU                 57    0.188  28.865  -0.181   0.021   0.756   0.223
B F  1DANU                 58    0.228  45.659  -0.550   0.021   0.451   0.528
E G  1DANU                 59    0.485  38.209  -0.442   0.004   0.085   0.910
E K  1DANU                 60    0.552 113.588  -1.009   0.005   0.015   0.979
E D  1DANU                 61    0.580  83.607  -0.067   0.004   0.085   0.910
B L  1DANU                 62    0.093  16.955   0.149   0.004   0.616   0.381
E I  1DANU                 63    0.279  51.615   1.080   0.001   0.900   0.099
B Y  1DANU                 64    0.060  12.865   0.662   0.001   0.959   0.040
B T  1DANU                 65    0.217  30.126   1.636   0.000   0.983   0.017
B L  1DANU                 66    0.026   4.852   1.022   0.000   0.983   0.017
B Y  1DANU                 67    0.118  25.217   1.606   0.000   0.983   0.017
B Y  1DANU                 68    0.060  12.758   0.643   0.000   0.983   0.017
B W  1DANU                 69    0.152  36.580   0.866   0.000   0.983   0.017
E K  1DANU                 70    0.366  75.348   0.699   0.002   0.816   0.182
E S  1DANU                 71    0.480  56.303  -0.346   0.004   0.197   0.799
E S  1DANU                 72    0.548  64.261  -0.200   0.005   0.045   0.951
E S  1DANU                 73    0.510  59.772  -0.392   0.019   0.141   0.840
E S  1DANU                 74    0.659  77.282   1.163   0.064   0.216   0.721
E G  1DANU                 75    0.473  37.194   0.126   0.074   0.484   0.442
E K  1DANU                 76    0.520 106.882   0.978   0.021   0.756   0.223
E K  1DANU                 77    0.329  67.737   0.890   0.011   0.918   0.071
E T  1DANU                 78    0.546  75.772   0.849   0.018   0.846   0.136
E A  1DANU                 79    0.361  39.815   0.083   0.021   0.756   0.223
E K  1DANU                 80    0.349  71.830   0.107   0.023   0.655   0.322
E T  1DANU                 81    0.400  55.452  -0.235   0.022   0.359   0.619
E N  1DANU                 82    0.667  97.693  -0.116   0.052   0.084   0.864
E T  1DANU                 83    0.390  54.024  -0.261   0.118   0.150   0.732
E N  1DANU                 84    0.389  57.008  -0.227   0.074   0.484   0.442
E E  1DANU                 85    0.340  59.468   0.261   0.085   0.775   0.140
B F  1DANU                 86    0.215  43.231   0.645   0.085   0.775   0.140
B L  1DANU                 87    0.128  23.492   0.975   0.169   0.612   0.219
E I  1DANU                 88    0.415  76.683   0.603   0.148   0.418   0.435
E D  1DANU                 89    0.489  70.537   0.016   0.066   0.296   0.638
B V  1DANU                 90    0.069  10.636   0.266   0.056   0.142   0.802
E D  1DANU                 91    0.461  66.401   1.019   0.018   0.088   0.893
E K  1DANU                 92    0.595 122.309   0.202   0.018   0.019   0.964
E G  1DANU                 93    0.490  38.579  -0.538   0.005   0.015   0.979
B E  1DANU                 94    0.192  33.595  -0.019   0.004   0.138   0.858
B N  1DANU                 95    0.255  37.288  -0.715   0.004   0.514   0.481
B Y  1DANU                 96    0.086  18.357   1.096   0.002   0.816   0.182
B C  1DANU                 97    0.037   5.195   0.160   0.001   0.900   0.099
B F  1DANU                 98    0.039   7.908   0.571   0.001   0.959   0.040
B S  1DANU                 99    0.179  20.955   1.095   0.001   0.959   0.040
B V  1DANU                100    0.027   4.181   0.834   0.001   0.959   0.040
B Q  1DANU                101    0.173  30.880   0.711   0.001   0.959   0.040
B A  1DANU                102    0.039   4.287  -1.076   0.001   0.959   0.040
B V  1DANU                103    0.119  18.213   0.390   0.001   0.900   0.099
B I  1DANU                104    0.095  17.575   0.659   0.003   0.718   0.279
B P  1DANU                105    0.188  26.706  -0.383   0.022   0.359   0.619
E S  1DANU                106    0.278  32.523  -0.595   0.019   0.141   0.840
E R  1DANU                107    0.438 100.233  -0.176   0.056   0.142   0.802
E T  1DANU                108    0.533  73.983  -0.991   0.118   0.150   0.732
E V  1DANU                109    0.368  56.592  -0.905   0.113   0.043   0.844
E N  1DANU                110    0.368  53.817  -1.323   0.053   0.043   0.903
E R  1DANU                111    0.403  92.287  -0.446   0.058   0.017   0.925
E K  1DANU                112    0.607 124.901  -0.451   0.018   0.019   0.964
B S  1DANU                113    0.226  26.440  -1.487   0.018   0.019   0.964
E T  1DANU                114    0.682  94.607  -0.418   0.018   0.019   0.964
E D  1DANU                115    0.455  65.623  -0.936   0.005   0.015   0.979
E S  1DANU                116    0.258  30.202  -0.439   0.005   0.015   0.979
E P  1DANU                117    0.598  84.885   0.761   0.018   0.088   0.893
E V  1DANU                118    0.416  64.001   0.352   0.005   0.336   0.660
E E  1DANU                119    0.434  75.820   1.320   0.004   0.616   0.381
B C  1DANU                120    0.151  21.228  -0.526   0.004   0.514   0.481
E M  1DANU                121    0.615 123.062  -0.846   0.005   0.015   0.979

Explain the output.  Go back.
```

---
